# Supplementary material for: The interactome of intact mitochondria by cross-linking mass spectrometry provides evidence for coexisting respiratory supercomplexes
Source: Mol Cell Proteomics. 2017 Dec 8;17(2):216–32. doi: 10.1074/mcp.RA117.000470 (PMC5795388; doi:10.1074/mcp.RA117.000470)
Supplement: Supplemental Data [file supp_RA117.000470_133922_0_supp_23978_fzffwf.zip › spectra_annotation/mito_spectra_annotation/223-1-4-1-18-1.pdf]

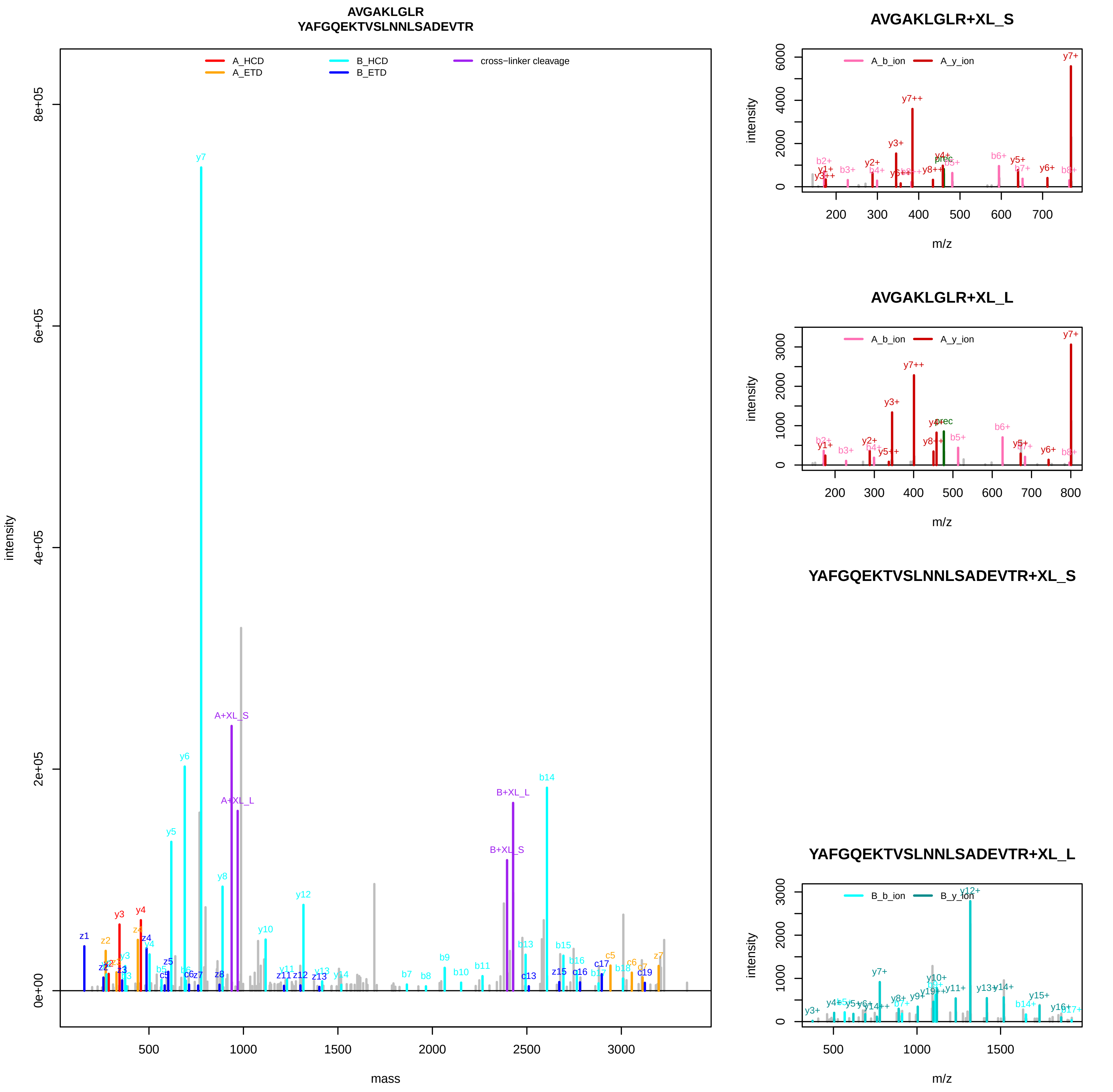

AVGAKLGLR
YAFGQEKTVSLNNLSADEVTR
A_HCD
A_ETD
B_HCD
B_ETD
cross−linker cleavage
intensity
mass
0e+00
2e+05
4e+05
6e+05
8e+05
500
1000
1500
2000
2500
3000
y7
A+XL_S
A+XL_L
B+XL_L
B+XL_S
b14
y6
y5
y8
y12
y10
y3
y4
z1
z2
z4
c5
c6
z7
b9
b11
b13
b15
b16
y3
c13
c16
c17
c19
AVGAKLGLR+XL_S
A_b_ion
A_y_ion
intensity
m/z
y7+
y7++
y3+
y2+
y4+
prec
b6+
y5+
y6+
b8+
AVGAKLGLR+XL_L
YAFGQEKTVSLNNLSADEVTR+XL_S
YAFGQEKTVSLNNLSADEVTR+XL_L
B_b_ion
B_y_ion
y12+
y7+
y10+
y11+
y13+
y14+
y15+
y16+
